# In Silico Analysis of Huntingtin Homologs in Lower Eukaryotes

**DOI:** 10.3390/ijms22063214

**Published:** 2021-03-22

**Authors:** Valentina Brandi, Fabio Polticelli

**Affiliations:** 1 Department of Sciences, Roma Tre University, 00146 Rome, Italy; valentina.brandi@uniroma3.it; 2 National Institute of Nuclear Physics, Roma Tre Section, 00146 Rome, Italy

**Keywords:** huntingtin, molecular modelling, function prediction, *Caenorhabditis elegans*

## Abstract

Huntington’s disease is a rare neurodegenerative and autosomal dominant disorder. HD is caused by a mutation in the gene coding for huntingtin (Htt). The result is the production of a mutant Htt with an abnormally long polyglutamine repeat that leads to pathological Htt aggregates. Although the structure of human Htt has been determined, albeit at low resolution, its functions and how they are performed are largely unknown. Moreover, there is little information on the structure and function of Htt in other organisms. The comparison of Htt homologs can help to understand if there is a functional conservation of domains in the evolution of Htt in eukaryotes. In this work, through a computational approach, Htt homologs from lower eukaryotes have been analysed, identifying ordered domains and modelling their structure. Based on the structural models, a putative function for most of the domains has been predicted. A putative *C. elegans* Htt-like protein has also been analysed following the same approach. The results obtained support the notion that this protein is a orthologue of human Htt.

## 1. Introduction

Human huntingtin (HsHtt) is a huge, 3144 amino acids long protein. An autosomal dominantly inherited expansion of the CAG repeats on the first exon of the protein gene results in the production of a mutant Htt with an abnormally long polyglutamine (polyQ) tract that leads to Huntington’s disease (HD), a severe, deadly neurodegenerative disease [1]. The protein is well conserved from flies to mammals, the highest sequence identity being observed between mammalian homologs. On the contrary, the polyQ tail is not conserved, suggesting that it may play a role in the precise modulation of the protein’s functions as a result of recent evolutionary achievements [2]. The N-terminal region of the protein includes the polyQ stretch, which starts at the amino acid 18, followed by a proline-rich domain (PRD) found only in mammals, indicating a recent evolution of the Htt protein [1,2].

Downstream of the polyQ, there are several HEAT repeats, 40 amino acids long structural motifs that consist of two antiparallel α-helices linked by a short loop. The HEAT repeats are packed together to form a flexible rod (denoted α-rod) and can act as a scaffold for diverse protein complexes and mediate inter- and intra-molecular interactions [2,3,4,5].

The comparison of Htt homologs can help to understand if there is a functional conservation of domains in the evolution of eukaryotes. In the Vertebrata subphylum Htt homologs are highly conserved (>80% sequence identity) [6]. Instead, the only entirely known amino acid sequence among invertebrates is the one from *Drosophila melanogaster*, in the protostome branch. This is characterized by an additional region and five conserved regions (20–50% sequence identity with the human protein) distributed throughout the length of the protein. These regions may represent a remnant of the ancestral Htt at the origin of the Protostomia-Deuterostomia branches [6].

**Figure 1:**
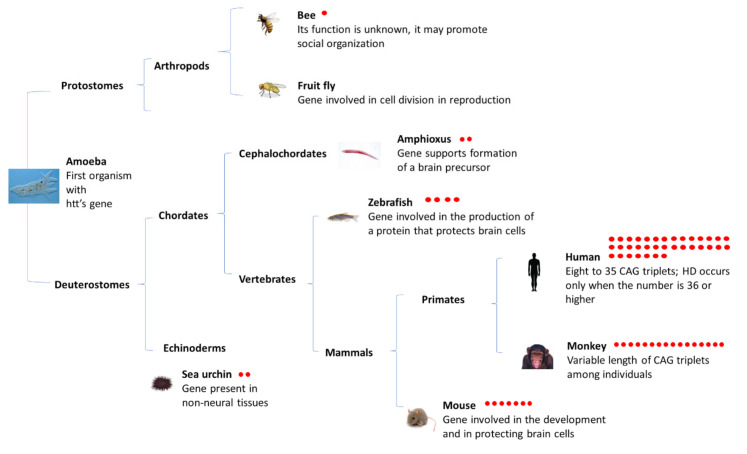
Evolution of HTT gene and CAG triplets. *Dictyostelium discoideum* amoebas are the most ancient organisms to carry the HTT gene, but without CAG triplets in the same position of the human counterpart. The gene regulates a number of vital cellular processes, including the transition of *D. discoideum* to its multicellular stage. The amoeba preceded the division of the tree of life into its two branches more than 550 million years ago: the protostomes, which include insects, crustaceans and mollusks, and the deuterostomes, which led to the first vertebrates, fishes, birds, amphibians, reptiles, mammals, primates and modern human beings. Only the deuterostomes went on to accumulate CAG triplets at the same place in which, in the human gene, occurs the mutation that causes HD. HTT gene starts to accumulate CAG triplets in the echinoderms, where two triplets are found. Further, two triplets occur in the sequence of amphioxus. CAG triplets begin to lengthen appreciably in organisms with a more sophisticated nervous systems. The number of red dots indicates the number of CAG triplets [6,7].

Htt is present in a lower complexity deuterostome, the tunicate *Halocynthia roretzi* (sea pineapple), and in the echinoderm *Heliocidaris herithrogramma* (sea urchin), but *Dictyostelium discoideum*, an amoeba, was the first organism known to carry the gene in a form that is slightly different from the human version (Figure 1) [6].

Andrade and colleagues have hypothesized the presence of Htt in the nematode *Caenorhabdtis elegans*, but not in *Saccharomyces cerevisiae*, thus confirming that HTT is, from an evolutionary viewpoint, an ancient gene [5].

HTT starts to acquire CAG triplets in a category of basal deuterostomes called echinoderms (such as the sea urchin *Strongylocentrotus purpuratus*), where two CAG triplets in the initial part of the gene have been identified [2] (Figure 1). Notwithstanding the presence of a primitive nervous system in sea urchins, the gene is present mainly in non-neural tissues, indicating that early on in evolution, the gene with its two CAG triplets did not play a crucial role in the nervous system. Research on the triplets in protostomes indicates that they are uncommon [7]. The analyses of the DNA sequences in the deuterostomes HTT revealed, as for sea urchins, that two CAG triplets occur in the sequence of the amphioxus, of the Cephalochordata family [7]. However, in amphioxus, the nucleotide sequence around the triplet pair is similar to that in vertebrates, including humans, and the protein encoded by the gene is largely confined to neural tissues. This may have contributed to the formation of the primitive brain. In fact, the CAG triplets tend to increase in organisms with a more complex nervous systems, until they reach their maximum extension in humans [7].

The study of new animal models opens the possibility of a better understanding of the evolution and functions of Htt. Therefore, in this work, the domain composition of Htts present in the ancient amoeba *Dictyostelium discoideum* and in the basal chordates *Ciona intestinalis* and *Branchiostoma floridae* has been investigated. Moreover, the possibility that Htt-like proteins are present also in lower complexity organisms has been probed focussing on the nematode *Caenorabditis elegans*.

*Dictyostelium discoideum* is a good model organism used in cell and developmental biology studies for its simple life cycle [8]. In addition, in the last few years, it has been used for the study of human diseases and the evaluation of drug effects [9].

The *Dictyostelium* genome possesses a single gene coding for Htt (dictyBase ID: DDB_G0272344). It is located on chromosome 2 and consist of four exons [10]. Although *Dictyostelium* naturally encodes proteins that in other organisms lead to the formation of toxic aggregates, it has the ability to withstand aggregation of proteins with long polyglutamine stretches. However, how this occurs is completely unknown [11].

*Dictyostelium* Htt (DdHtt) includes a stretch of polyglutamine (19 residues long) as well. Unlike HsHtt, this stretch is encoded by the trinucleotide repeat CAA and the protein lacks the subsequent polyproline (polyP) domain. In addition, it is located further downstream of the initial methionine as compared to the human protein.

Studies concerning the function of Htt in *Dictyostelium* have shown that it is involved in the synchronous development of the organism cells and in the actin cytoskeleton-membrane dynamics related to the cell shape [10], it is necessary for cytokinesis and chemotaxis and for the preservation of cellular integrity under osmotic stress conditions [10].

*Ciona intestinalis* is a tunicate (sea squirt) belonging to the phylum Chordata, which has a great relevance in evolutionary studies because it has the advantage of being a chordate-invertebrate. Indeed, like Chordata, it shows a body plan and an embryonic development very similar to those of vertebrates [12] but, like invertebrates, it displays enough genetic divergence from vertebrates to allow evolutionary and comparative analyses at the protein level. Thus, the large evolutionary distance separating tunicates and vertebrates (about 520 million years) [13] could allow the identification of a Htt “signature” related to the ancestral function(s) of the gene/protein in Chordata [14].

*Branchiostoma floridae* is a lancelet (also known as amphioxus) that belongs to the subphylum Cephalochordata of the phylum Chordata. The nervous system development of the amphioxus is particularly close to that of vertebrates as it includes vertebrate-like anatomical characteristics but lacks the typical subcellular and tissue specialization of the vertebrates nervous system [15]. Thus, this organism is particularly useful to deduce features already present in the last common ancestor of chordates.

The amphioxus Htt (BfHtt) comes from an invertebrate chordate whose phylogenetic node of divergence is thought to go back 540 million years, while *Ciona intestinalis* seems to have diverged more recently [16].

BfHtt is mainly abundant in the neural compartment, indicating that Htt, in amphioxus, could be involved in neuronal functions [15].

BfHtt has two glutamine residues (Q17 and Q18) in the same polyQ tract position of HsHtt (Figure S1), thus suggesting that a polyQ tract was emerging already 540 million years ago [15] in a non-vertebrate species (Figure 1).

The differences in the length of the polyQ tract between amphioxus and vertebrates suggest that Htt may have evolved different biochemical properties in both lineages [15].

One further characteristic of BfHtt is the complete absence of the polyP-rich region, such as in ascidian Htt; indeed the polyP tract is present only in the mammalian proteins (Figure S1). On the contrary, the first 17 amino acids of BfHtt, with its three lysine residues involved in the intracellular distribution of the protein between the cytoplasm and nucleus in vertebrates, are also strongly conserved in HsHtt (Figure S1). With respect to the latter, conservation of the amino acid sequence in the amphioxus protein (46%) is higher than in that of *C. intestinalis* protein (34%) (Table S1). The comparison of the gene structure of BfHtt with the human and ascidian homologues highlights that amphioxus Htt is closer to vertebrates Htt than the ascidian one and leads to the hypothesis that its functions are also possibly closer to those of the vertebrates protein [15].

Almost all HEAT repeats in amphioxus (Table 1, Figure S1) seem to be conserved in the human homolog, an exception being the last HEAT consensus at 3020–3038 in amphioxus sequence that has no correspondence in the human protein.

*Caenorhabditis elegans* is a nematode that lives in temperate soil environments and is one of the “supermodels” of modern biology, as it possesses many genes with a significant similarity to those involved in human diseases.

Here, the same bioinformatics approach used to analyse HsHtt sequence [17] before the structure was determined by cryo-electron microscopy [18] has been applied to the Htt sequence of the model organisms *Dictyostelium discoideum*, *Ciona intestinalis* and *Branchiostoma floridae*. Further, following the same approach, an Htt-like protein has been for the first time identified in *Caenorhabditis elegans* and its structural features have been predicted and analysed.

## 2. Results

### 2.1. Analysis of Dictyostelium discoideum Htt

**Figure 2:**
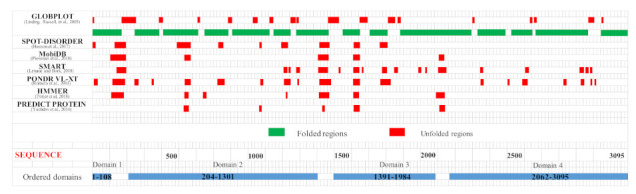
Predicted ordered (green) and disordered (red) regions of *Dictyostelium* homologue of HsHtt. Htt domains predicted as ordered by consensus among the different disorder prediction methods are indicated in the bottom part of the figure as light blue bars.

Dictyostelium Htt (DdHtt) displays a sequence length of 3095 amino acids, similar to that of the human counterpart (29% sequence identity, Table S1), and has no significant sequence similarity with other Dictyostelium proteins. Analysis of the sequence order/disorder of DdHtt indicates that the protein is characterized by four ordered domains (hereafter named domain 1–4, Figure 2), as compared to the five ordered domains characterizing the human counterpart [17,18].

**Figure 3:**
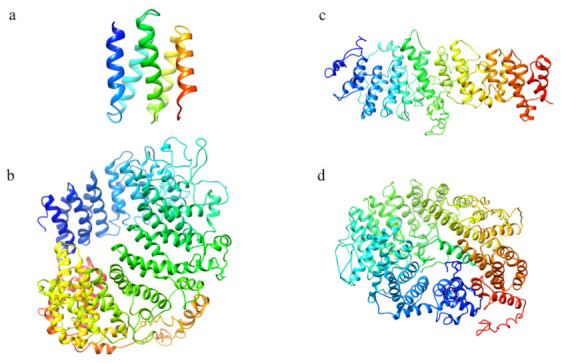
Structural models of the ordered domains of *Dictyostelium* homologue of Htt. (**a**) Domain 1; (**b**) Domain 2; (**c**) Domain 3; (**d**) Domain 4.

**Figure 4:**
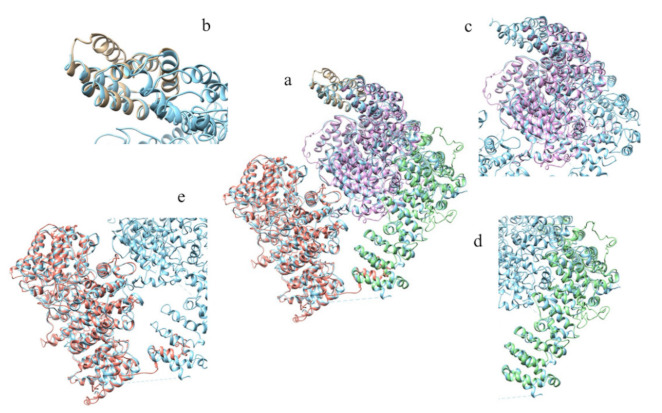
Comparison between the structural models of DdHtt and the three-dimensional structure of HsHtt. (**a**) Superimposition between the models of the four ordered domains of DdHtt (domain 1 in tan, domain 2 in pink, domain 3 in salmon, domain 4 in green) and the structure of the human protein (in sky blue, PDB code: 6EZ8 [18]). Side panels show views of the single domains (domain 1 in (**b**), domain 2 in (**c**), domain 3 in (**d**), and domain 4 in (**e**)).

The structure prediction of the ordered domains (Table S2) shows the typical conformation of HEAT repeats, as depicted in Figure 3. In particular, the domains 2, 3, and 4 have structural similarity with karyopherins, proteins involved in nuclear import of several cargoes (Tables S4–S7), as has been observed for HsHtt ordered domains [17]. Further, the structural models of the DdHtt domains cover almost the entire structure of HsHtt (Figure 4), indicating a substantial conservation of the overall Htt structure.

**Figure 5:**
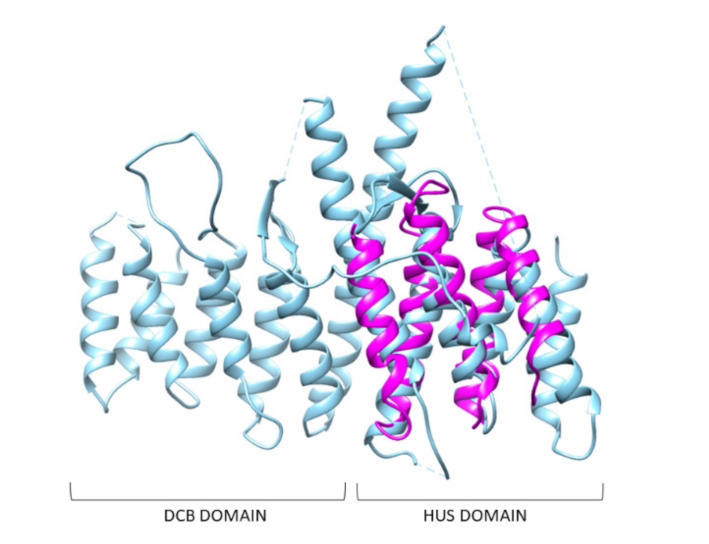
Comparison between the structural model of domain 1 of DdHtt and the three-dimensional structure of DCB-HUS. Superimposition between domain 1 of DdHtt (in magenta) with the N-terminal DCB-HUS domain of *Thermothielavioides terrestris* Sec7 (in cyan) (PDB code: 5HAS [19]).

Domain 1 displays structural similarity (Table S4) with the N-terminal DCB-HUS domain of Thermothielavioides terrestris Sec7 (PDB code: 5HAS [19] Figure 5), an Arf guanine nucleotide exchange factor (Arf-GEF) localized at the trans-Golgi network (TGN) (Richardson et al. 2016). The ARF family GTPases are characterized by an N-terminal extension of ~14 amino acids and covalent modifications at or near this end with regard to the other families of small, regulatory GTPases (RAS, RHO, RAB). The ARFs are involved in recruitment of coat proteins/complexes and initiation of vesicle formation in membrane trafficking, particularly at the Golgi [20]. ARFs require GEFs to accelerate nucleotide exchange. All ARF GEFs share a common catalytic ~200-residue Sec7 domain (Sec7d) and a common mechanism of action to promote nucleotide exchange, but display diversity in their actions and regulation in cells [20].

Unfortunately, residues involved in vesicle trafficking are unknown. However, since domain1 overlaps with HUS domain, it could be involved in vesicles trafficking at the trans-Golgi network.

**Figure 6:**
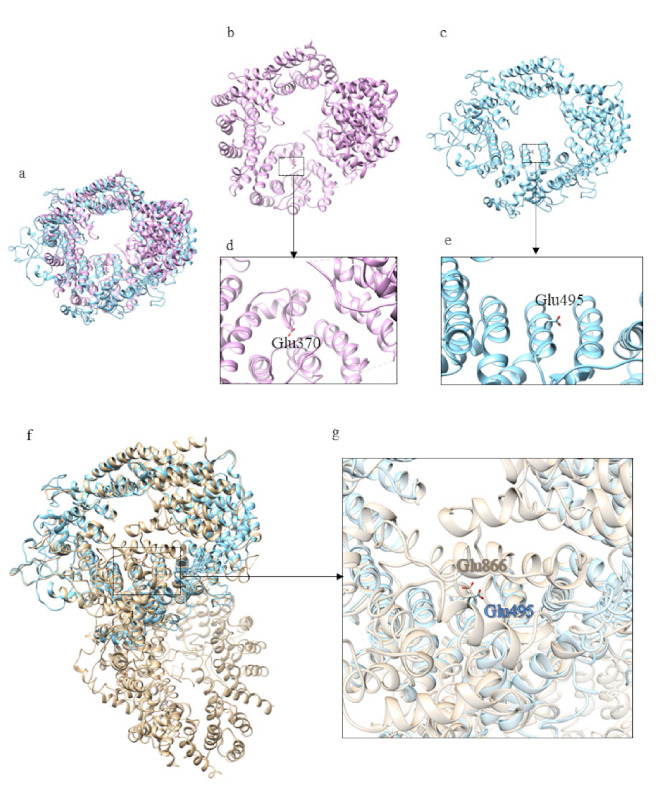
Comparison between the structural model of domain 2 of DdHtt and the three-dimensional structure of Cse1. (**a**) Superimposition between domain 2 of DdHtt (in sky blue) and Cse1 in its cargo-free conformation (in pink, PDB code: 1Z3H [21]); (**b**) structural model of domain 2 of DdHtt; (**c**) structure of Cse1, in (**d**,**e**) details of the DdHtt domain 2 and Cse1, respectively; (**f**) superimposition between human Htt structure (in tan) and domain 2 of DdHtt (in sky blue); (**g**) detail of the residue hypothetically involved in Ran binding, in DdHtt domain (in tan) and in human Htt (in sky blue).

The closest structural homolog of the domain 2 is exportin Cse1 in its cargo-free conformation (PDB code: 1Z3H [21]) (Table S5). Although there isn’t a complete superimposition between the model of domain 2 and the structure of the exportin, the fold is very similar. RanGTP interacts with Cse1 at several distinct sites. In cargo-free Cse1, most of these residues are occluded and the intramolecular interaction between the N- and C-terminal prevents cargo binding in the absence of RanGTP. The acidic residue directly involved in Ran binding is the invariant Glu370 and it is also conserved in the model of domain 2 (Figure 6c) and in human Htt (Figure 6g). This suggests that domain 2 of *Dictyostelium* Htt is involved in protein transport, consistent with one of the functions of HsHtt.

Among the structural homologs of DdHtt domain 3 (Table S6) there are two exportins (PDB codes: 5DLQ [22]; 3A6P [23]), but the two structural homologs are larger than domain 3, and the residues involved in the binding of RanGTP are not conserved. Thus, a function for this domain has been difficult to hypothesize, even if the fold of this domain resembles that of karyopherins, as it has been observed for the orthologous domain of HsHtt [17,18].

**Figure 7:**
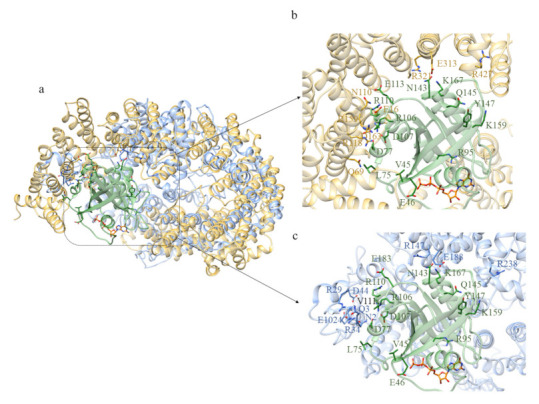
Comparison between the structural model of domain 4 of DdHtt and the three-dimensional structure of Exp-5. (**a**) Superimposition between the structural model of DdHtt domain 4 (in sky blue) and Exp-5 structure (PDB code: 3A6P [23], in yellow), in complex with RanGTP (in green, GTP in orange). In (**b**) residues involved in the interaction between RanGTP and Exp-5, and in (**c**) the conserved residues in DdHtt domain 4.

Domain 4 of DdHtt displays structural similarity (Table S7) with Exportin-5 (Exp-5) (PDB code: 3A6P [23]), a member of the pre-microRNA nuclear export machinery [23]. Even if domain 4 has a more closed fold with respect to Exp-5 (Figure 7a), some of the residues involved in the interaction with RanGTP are conserved (Figure 7b,c). Thus, domain 4 could be involved in the nuclear export of micro-RNAs as well.

### 2.2. Analysis of Ciona intestinalis Htt

The Htt protein of *C. intestinalis* (CiHtt) is 2945 amino acids long, notably shorter than its vertebrate homologs, which are 3130 amino acids long on average. This difference in length is apparently due to deletions in the N-terminal region of the protein (Figure S1). Moreover, the N-terminal region of the C. intestinalis protein lacks the polyQ domain, or any kind of simple repeat (Figure S1). Even the proline-rich region typical of mammalian Htts is absent. However, the amino acid sequence identity between human and ascidian Htt is 34% (Table S1). A total of 8 HEAT repeats are present in the ascidian Htt. These are located as tandem arrays or as single elements in the N-terminal (4 repeats), central (2 repeats), and C-terminal (2 repeats) regions [1,14] (Table 1).

The analysis of Htt proteins multiple sequence alignment (Figure S1) shows that all ascidian Htt HEAT repeats are conserved in the human ortholog.

**Figure 8:**
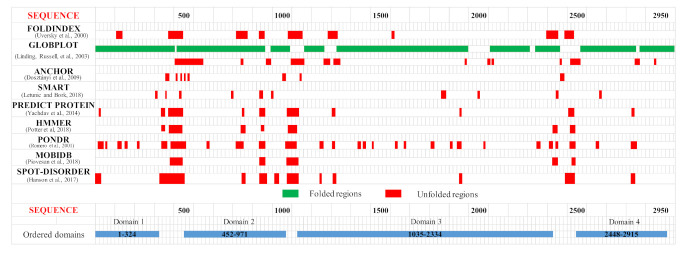
Predicted ordered (green) and disordered (red) regions of ascidian Htt. Htt domains predicted as ordered by consensus among the different methods are indicated in the bottom part of the figure as light blue bars.

**Figure 9:**
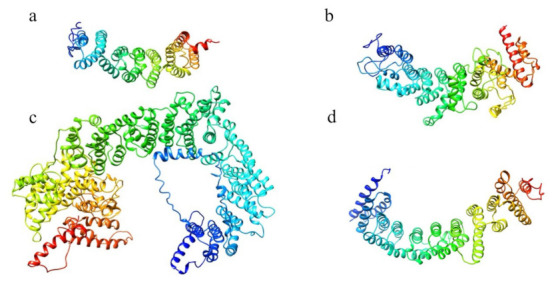
Structural models of the ordered domains of CiHtt. Domain 1 in (**a**), domain 2 in (**b**), domain 3 in (**c**) and domain 4 in (**d**).

An identification and analysis of the ordered domains similar to that performed on HsHtt has also been carried out for the ascidian Htt sequence (Figure 8, Table S8), leading to the identification of four ordered domains. Then, the structure prediction (Table S9) has revealed that all the models display an α-helical structure (Figure 9), due to the presence of HEAT repeats. Domain 1, 2, and 4 are characterized by a concave shape. Instead, the third ordered domain exhibits structural similarity with the third ordered domain identified in HsHtt [17,18], including the presence of an α-helix stemming from the C-terminal region of the domain and interacting with a concave region on the opposite side (Figure 9c). All the models have shown structural similarity with HsHtt structure (Tables S10–S13).

**Figure 10:**
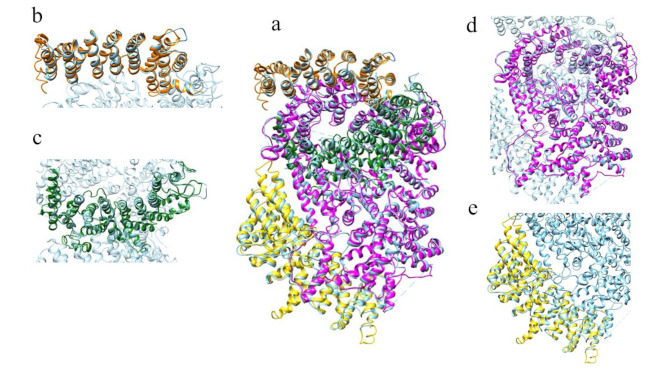
Comparison between the structural models of CiHtt and the three-dimensional structure of HsHtt. (**a**) Superimposition between the models of the four ordered domains of ascidian Htt (domain 1 in orange, domain 2 in dark green, domain 3 in magenta, domain 4 in yellow) and the structure of the human protein (in sky blue, PDB code: 6EZ8 [18]). In the side panels are shown views of the single domains (domain 1 in (**b**), domain 2 in (**c**), domain 3 in (**d**), and domain 4 in (**e**)).

Figure 10 displays the superimposition between the models of the ordered domains of the ascidian protein and the corresponding domains of HsHtt. All the models (Figure 10b–d) overlap with different regions of the human protein, covering almost the entire structure, even though the ascidian protein is slightly shorter. Given the sequence identity (34%) and the structural homology between the models of the ordered domains of ascidian Htt and the human structure, the prediction of the presence in the ascidian protein of four ordered domains and the fold of the corresponding structural models appear reliable.

**Figure 11:**
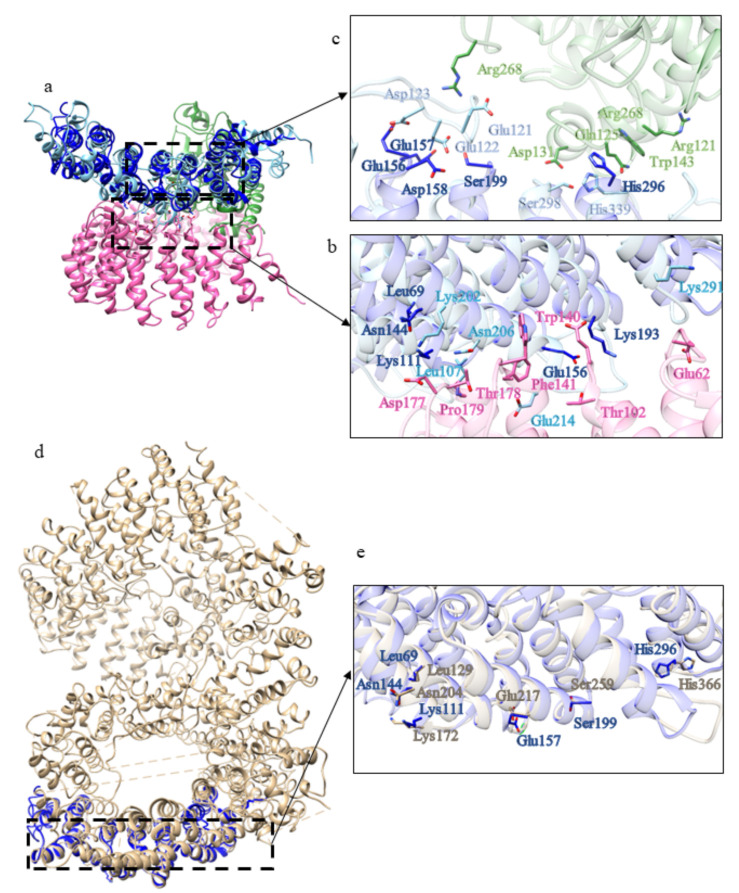
Comparison between the structural model of domain 1 of CiHtt and the three-dimensional structure of protein phosphatase 2A. (**a**) Superimposition between domain 1 of ascidian Htt (depicted in blue) and the regulatory B subunit (in sky blue) of the protein phosphatase 2A (PDB code: 2IAE, subunit A in pink, subunit C in green, [24]). The residues in the interface with A and C subunits are shown in (**b**,**c**), respectively; (**d**) superimposition between human Htt (in tan) and domain 1 of ascidian Htt (depicted in blue); (**e**) some of the residues involved in the interactions between A and C subunits are conserved in HsHtt.

The first domain displays structural similarity (Table S10) with the human serine/threonine-protein phosphatase 2A (PP2A) 56 kDa regulatory subunit (PDB code: 2IAE [24]). Indeed, PP2A is a holoenzyme and the core enzyme is made up of a scaffolding A subunit and a C subunit. The binding of a regulatory B subunit to the AC core enzyme regulates PP2A activities. The methylation of the carboxylate group of the C-terminal residue Leu 309 of C subunit facilitates the recruitment of the regulatory B subunit to the AC core dimer. Some of the residues involved in the interactions between A and C subunits are preserved in domain 1 of *C. intestinalis* (Figure 11b,c) and HsHtt (Figure 11e). The interface between A and B subunits is relatively loose (Figure 11b), due to the fact that A and B subunits do not form a stable complex. The weak binding between A and B subunits is enhanced by binding of the methylated C-terminal tail to this interface [24].

The acidic cluster in the long intra-repeat loop 2 of B subunit, which interacts through salt bridges with R268 in the C subunit, is also preserved in domain 1 of ascidian Htt (Figure 11c).

**Figure 12:**
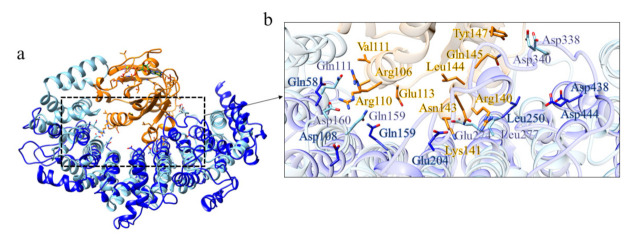
Comparison between the structural model of domain 2 of CiHtt and the three-dimensional structure of Impβ in complex with RanGTP. (**a**) Superimposition between the complex formed by Impβ (in sky blue, PDB code: 1IBR [24]) with RanGTP (in orange) and domain 2 of CiHtt (in blue). (**b**) Detail of the residues involved in the binding of RanGTP by Impβ and similar residues present of the corresponding region of domain 2 of CiHtt.

Among the structural homologs of domain 2 (Table S11) there is Importin β (Impβ) (PDB code: 1IBR [25]), a major mediator of nuclear protein import through the interaction with RanGTP. The conservation in CiHtt domain 2 of some residues involved in the binding of RanGTP by Impβ is shown in Figure 12. In agreement with Htt functions, this domain could be involved in proteins transport from cytoplasm to nucleus.

**Figure 13:**
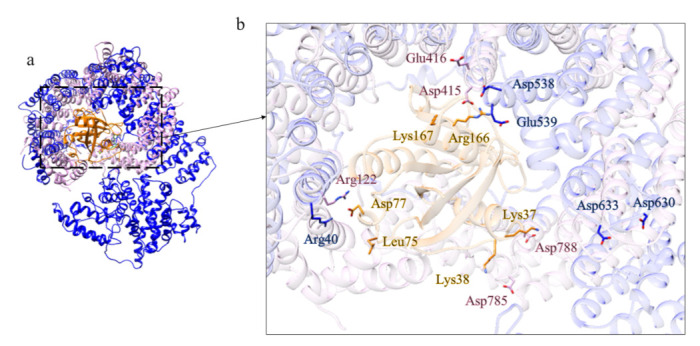
Comparison between the structural model of domain 3 of CiHtt and the three-dimensional structure of Importin 13 in complex with RanGTP. (**a**) Superimposition between domain 3 of CiHtt (in blue) and Importin 13 (PDB code: 2X1G [26]; in pink) in complex with RanGTP (in orange). (**b**) Detail of the residues involved in the interaction between Importin 13 and RanGTP and similar residues of domain 3 of CiHtt observed in the putative interacting region.

The structural model of domain 3 of CiHtt displays similarity with Importin 13 three-dimensional structure (Table S12) (PDB code: 2X1G [26]). Importin 13 is a bidirectional karyopherin that can mediate both import and export of cargoes [27]. Although the structure of the domain 3 has an additional portion, compared to Importin 13, residues potentially involved in the interaction with RanGTP are observed in the putative interaction region of domain 3 (Figure 13). Thus, this domain could be involved in cargoes transport, both in import and in export.

**Figure 14:**
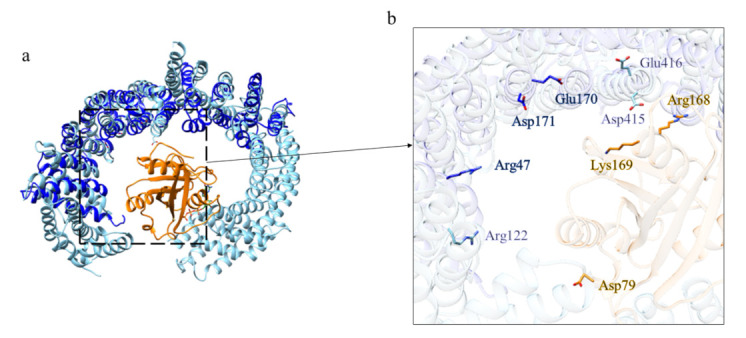
Comparison between the structural model of domain 4 of CiHtt and the three-dimensional structure of Importin 13 in complex with RanGTP. (**a**) Superimposition between domain 4 of CiHtt (in blue) and Importin 13 (PDB code: 2XWU [27], in sky blue); RanGTP is depicted in orange. (**b**) Details of the preserved residues involved in the interaction between Importin 13 and RanGTP.

In addition, the structural model of CiHtt domain 4 displays similarity (Table S13) with Importin 13 (PDB code: 2XWU [27]). The model of domain 4 of CiHtt is shorter than Importin 13 (Figure 14a), as already highlighted for domain 3. Residues that could be involved in the interaction with RanGTP are observed in the putative interaction region of domain 4 (Figure 14b), though not in orthologous position with respect to those mediating Importin 13-RanGTP interaction. Therefore, in the case of domain 4, a reliable prediction of its function is not possible.

### 2.3. Analysis of Branchiostoma floridae Htt

**Figure 15:**
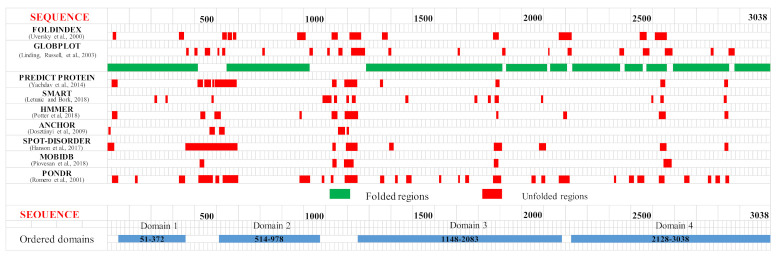
Predicted ordered (green) and disordered (red) regions of BfHtt. Htt domains predicted as ordered by consensus among the different methods are indicated in the bottom part of the figure as light blue bars.

The analysis of BfHtt sequence (Table S14) has led to the identification of four ordered domains, depicted in Figure 15.

**Figure 16:**
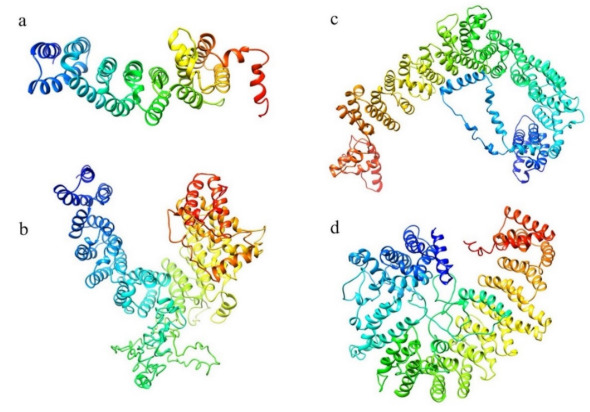
Structural models of the ordered domains of BfHtt. Domain 1 (**a**), domain 2 (**b**), domain 3 (**c**), domain 4 (**d**).

The structural models of the four ordered domains, obtained through I-TASSER (Table S15), show the typical topology of HEAT repeats (Figure 16) and structural similarity with HsHtt (Tables S16–S19). Interestingly, domain 3 has structural similarity with the human hunt3 domain (Figure 16c), as has been observed for CiHtt.

**Figure 17:**
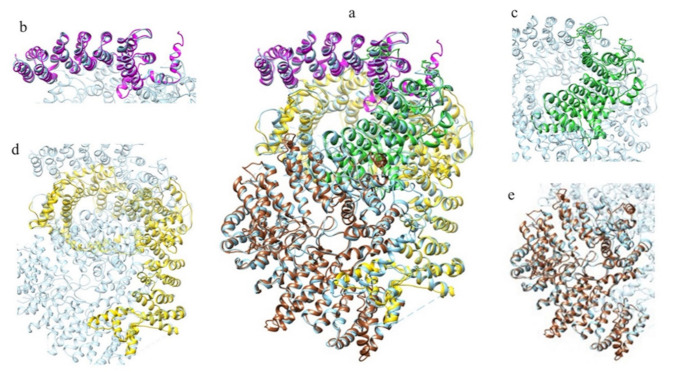
Comparison between the structural models of BfHtt and the three-dimensional structure of HsHtt. (**a**) Superimposition between the models of the four ordered domains of BfHtt (domain 1 in magenta, domain 2 in dark green, domain 3 in yellow and domain 4 in sienna) and the structure of human protein (in cyan, PDB code: 6EZ8 [18]). In the side panels are shown views of the single domains (domain 1 in (**b**), domain 2 in (**c**), domain 3 in (**d**), and domain 4 in (**e**)).

Figure 17 displays the superimposition between the models of the ordered domains of BfHtt and the human ones.

**Figure 18:**
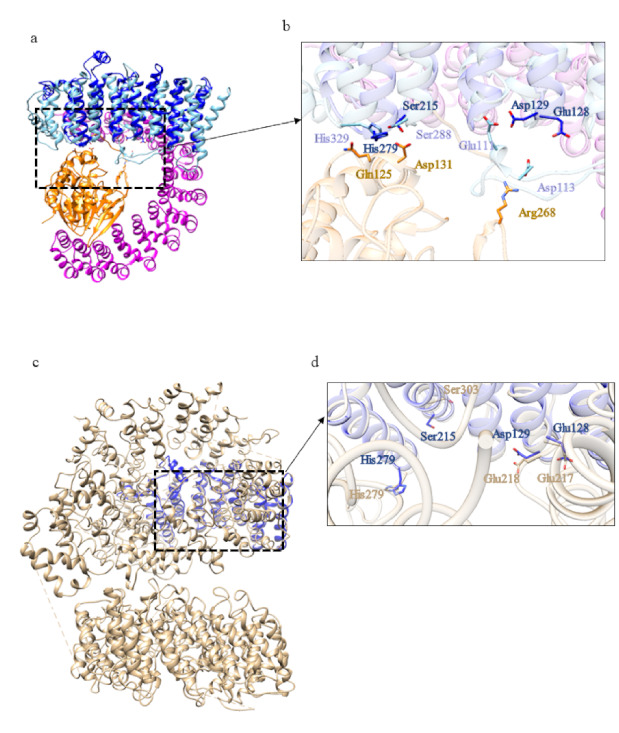
Comparison between the structural model of domain 1 of BfHtt and the three-dimensional structure of the regulatory subunit of the protein phosphatase 2A. (**a**) Superimposition between domain 1 of BfHtt (depicted in blue) and the regulatory subunit (in sky blue) of the protein phosphatase 2A (PDB code: 3FGA [28], subunit A in pink, subunit C in orange). The residues in the interface with C subunit are shown in panel (**b**). (**c**) Superimposition between human Htt (in tan) and domain 1 of BfHtt (in blue); (**d**) some residues involved in the interaction between B and C subunits are conserved in HsHtt.

As for the ascidian Htt, the models of BfHtt domains cover almost the entire structure of HsHtt, suggesting that the identification of the domains is rather reliable. Among the structural homologs of domain 1 (Table S16) there is the regulatory subunit of Serine/threonine-protein phosphatase 2A (PDB code: 3FGA [28]). As explained before, protein phosphatase 2A (PP2A), together with PP1, constitutes the major serine/threonine phosphatase in the cell and is involved in the control of a wide range of cellular processes [29]. In domain 1 of BfHtt and in HsHtt, some residues involved in the interaction between the PP2A B and C subunits are conserved (Figure 18), suggesting that this domain could contact A subunit in different ways.

**Figure 19:**
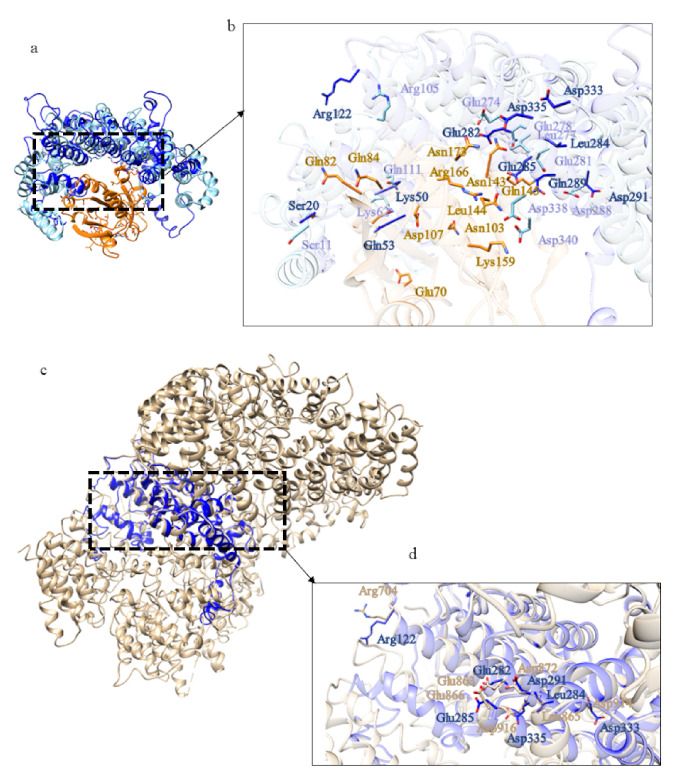
Comparison between the structural model of domain 2 of BfHtt and the three-dimensional structure of the complex Impβ-RanGTP. (**a**) Superimposition between the complex Impβ-RanGTP (in sky blue and orange, respectively; PDB code: 1IBR [25]) and the structural model of domain 2 of BfHtt (in blue). (**b**,**d**) details of the residues involved in the binding of RanGTP in domain 2 of BfHtt and HsHtt; (**c**) superimposition between HsHtt (in tan) and domain 2 of BfHtt (in blue).

BfHtt domain 2 displays structural similarity (Table S17) with Importin β (PDB code: 1IBR [25]) in complex with RanGTP, the same found for domain 2 of CiHtt. Interestingly, the structural model and the HsHtt display a distribution of charged residues very similar to that observed in the Impβ-RanGTP interface (Figure 19). Thus, BfHtt domain 2 could be involved in nuclear import of protein cargoes through interaction with RanGTP as well.

**Figure 20:**
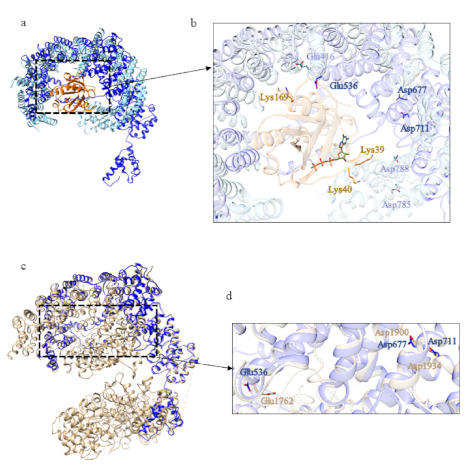
Comparison between the structural model of domain 3 of BfHtt and the three-dimensional structure of Imp13. (**a**) Superimposition between Imp13 (in sky blue, PDB code: 2XWU [27]; RanGTP in orange, GTP in green) and domain 3 of B. floridae (in blue). (**c**) superimposition between HsHtt (in tan) and domain 3 of BfHtt (in blue). (**b**,**d**) details of the residues involved in the binding of RanGTP in domain 3 of B. floridae (in blue) and in HsHtt (in tan).

One of the structural homologs of domain 3 (Table S18) is Importin13 (PDB code: 2XWU [27]). This is a peculiar β-karyopherin that can both import cargoes into the nucleus and export them out. In the cytoplasm, Imp13 binds a variety of different cargoes, among which Mago-Y14 and Ubc9, facilitating their import into the nucleus where association with RanGTP promotes their release [27]. Some of the residues involved in the binding of RanGTP are conserved in domain 3 of BfHtt (Figure 20b) and in HsHtt (Figure 20d). Thus, this domain could have a function as cargo transporter.

**Figure 21:**
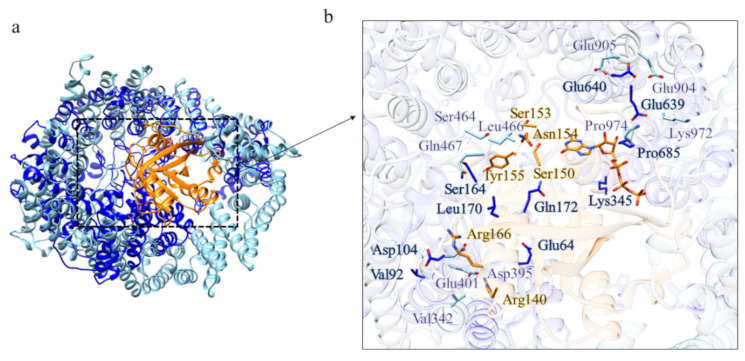
Comparison between the structural model of domain 4 of BfHtt and the three-dimensional structure of Imp13. (**a**) Superimposition between exportin Xpo4 (in sky blue, PDB code: 5DLQ [22], in complex with RanGTP, in orange) and domain 4 of BfHtt (in blue). (**b**) detail of the residues involved in the binding of RanGTP.

One of the structural homologs of domain 4 (Table S19) is the exportin Xpo4 in complex con RanGTP (PDB code: 5DLQ [22]). In domain 4, some of the residues involved in the interaction with RanGTP are conserved (Figure 21).

### 2.4. Analysis of a Putative Htt-Like Protein in Caenorhabditis elegans

**Figure 22:**
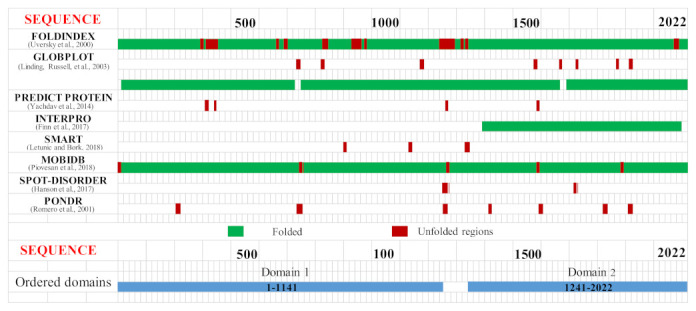
Predicted ordered (green) and disordered (red) regions of the putative *C. elegans* Htt-like protein. Htt domains, predicted as ordered by consensus among the different methods, are indicated in the bottom part of the figure as light blue bars.

No literature data are available on the existence of a Htt-like protein in *C. elegans*, even though the gene F21G4.6 is annotated as an ortholog of huntingtin. This gene codes for a protein of 2022 residues, which displays 21% sequence identity over a 13% of query coverage with HsHtt (Table S1, Figure S1). This sequence is shorter than the human one, due to a deletion observed in the N-terminal region. The uncharacterized protein sequence has been analysed adopting the same approach used to study human, amoeba, ascidian, and amphioxus Htt. In the *C. elegans* protein sequence, two putative ordered domains have been identified (Table S20, Figure 22).

**Figure 23:**
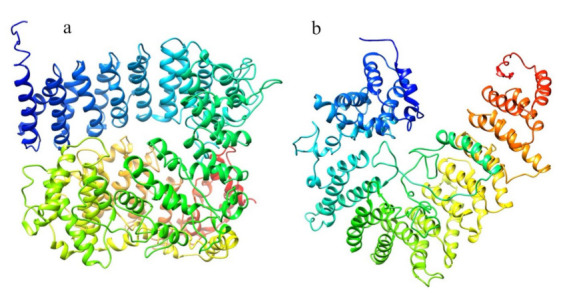
Structural models of the ordered domains of the Htt-like protein identified in *C. elegans*. (**a**) domain 1 and (**b**) domain 2.

**Figure 24:**
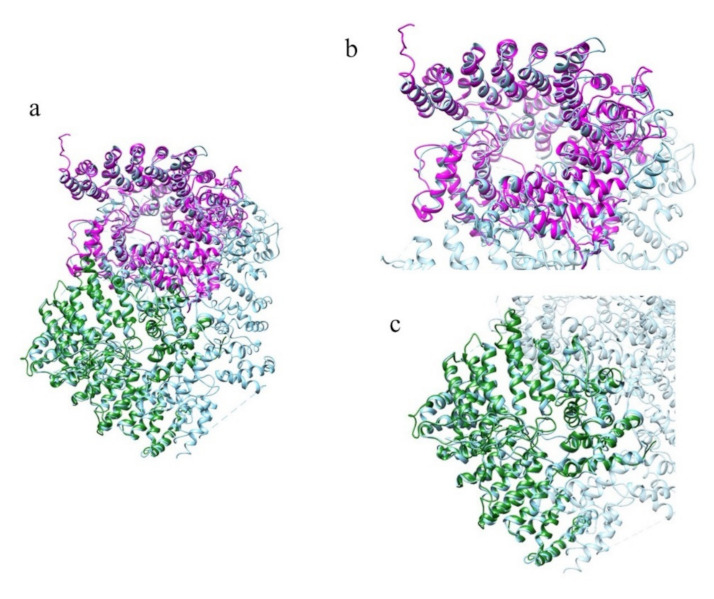
Comparison between the structural models of the Htt-like protein identified in *C. elegans* and the three-dimensional structure of HsHtt. (**a**) Superimposition between the models of the two ordered domains of the Htt-like protein (in magenta domain 1 and in dark green domain 2) identified in *C. elegans* and the structure of HsHtt (in sky blue, PDB code: 6EZ8 [18]). In the two side panels are shown views of the single domains (domain 1 in (**b**), domain 2 in (**c**).

The structure prediction (Figure 23, Table S21) has uncovered that both domains are made up of α-helices with a Karyopherin-like fold and have structural similarity with HsHtt (Figure 24, Tables S22 and S23). The first domain displays 38% sequence identity with the N-terminal region of HsHtt, corresponding to hunt1, hunt2 and a portion of the hunt3 ordered domains [17] (Table S1). Domain 2 displays 21% sequence identity with the C-terminal region of HsHtt, matching hunt5 ordered domain (Table S1), leaving the bridge region of human Htt structure uncovered (Figure 24). Indeed, HsHtt (PDB code 6EZ8) is the best structural homologue of both ordered domains of CeHtt (PDB code 6EZ8), with very high TM-score values (>0.8) and low RMSD values (≤2.0 Å).

The presence of Htt in the amoeba *Dictyostelium discoideum* has suggested that HTT is an old gene that has been lost in some evolutionary more recent animals, such as *C. elegans* [6]. The uncharacterized protein of *C. elegans* analyzed in this work could be a Htt-like protein that has lost two protein regions (the N-terminal and the corresponding “bridge region” of HsHtt).

**Figure 25:**
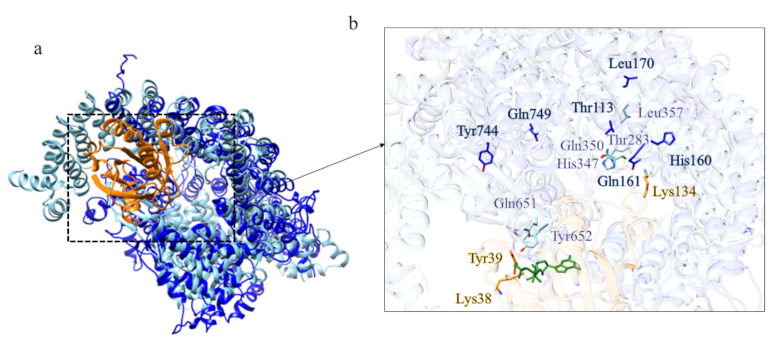
Comparison between the structural model of domain 1 of *C. elegans* putative Htt and Kap121p. (**a**) Superimposition between Kap121p (in sky blue, PDB code: 3W3T [30]; RanGTP in orange, GTP in green) and domain 1 of *C. elegans* putative Htt (in blue), (**b**) detail of the residues involved in the binding of RanGTP.

The first ordered domain displays structural similarity (Table S22) with the karyopherin Kap121p (PDB code: 3W3T [30]) of *Saccharomyces cerevisiae* in the cargo-free state. Even if domain 1 is shorter than Kap121p in the RanGTP binding region, some residues are conserved in the putative interaction interface (Figure 25).

**Figure 26:**
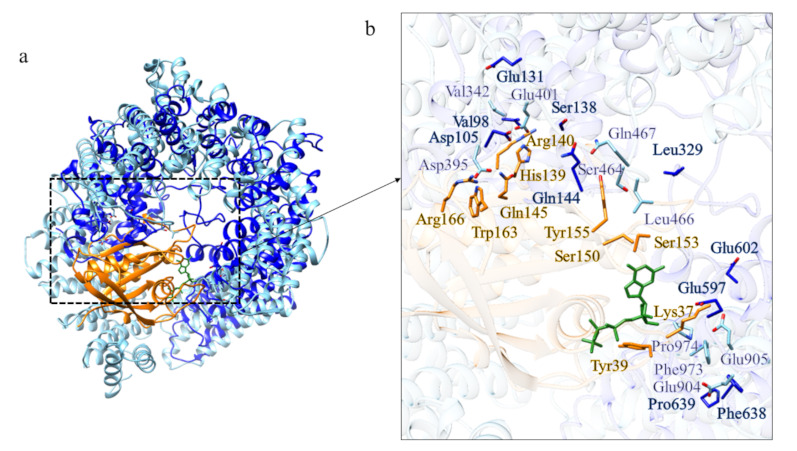
Comparison between the structural model of domain 2 of *C. elegans* putative Htt and Xpo4. (**a**) Superimposition between exportin Xpo4 (in sky blue, PDB code: 5DLQ [22], in complex with RanGTP in orange, GTP in green) and domain 2 of *C. elegans* (in blue), (**b**) detail of the residues involved in the binding of RanGTP.

Among the structural homologs of domain 2 (Table S23), there is the exportin Xpo4 in complex with RanGTP (PDB code: 5DLQ [22]), the same protein identified for domain 4 of B. floridae. In this case, the structure of the model of domain 2 is shorter than Xpo4; however, some of the residues involved in the binding of RanGTP are conserved (Figure 26).

## 3. Discussion

A structural analysis of ordered domains of Htt sequences has been performed in *D. discoideum*, *C. intestinalis*, *B. floridae* and *C. elegans*, through molecular modelling. A function for most of the ordered structural domains has been hypothesized by identifying the closest structural homolog of each domain (Table 2).

Domain1 of *D. discoideum* could have a role in vesicles trafficking at the trans-Golgi network. This domain has structural similarity with the N-terminal DCB-HUS domain of *Thermothielavioides terrestris* Sec7 (PDB code: 5HAS [19]), an Arf-GEF localized at the trans-Golgi network (TGN) [19]. TGN-localized Arf-GEFs are generally conserved in eukaryotes. Interestingly, mutations in the BIG2/ARFGEF2 human gene are associated with neurological disorders. Sec7 is a regulatory protein, which controls trafficking of vesicles that leave the Golgi complex. Sec7 is made up of different domains, such as “DCB” and “HUS”, two regulatory domains, whose specific functions are not known. However biochemical experiments have shown that the DCB/HUS domain helps Sec7 to insert another regulatory protein (called Arf1) into the membranes of the Golgi complex [19].

Domain 2 could be an exportin, since its structural homolog (Cse1, PDB code: 1Z3H [21]) mediates the recycling of importin α in the NLS (classical nuclear localization signal)-mediated pathway. Importin α binds both NLS-containing proteins and the receptor importin β in the cytosol. After the import complex is transported into the nucleus, both importin α and importin β are recycled back to the cytoplasm for a new cycle. Contrary to the importin β, importin α is exported in a ternary complex with Cse1 and RanGTP. The ternary export complex is separated in the cytoplasm where Ran-GTP is converted into Ran-GDP [21].

Domain 4 has shown structural similarity with an Exportin-5 (Exp-5, PDB code: 3A6P [23]), a member of the pre-microRNA nuclear export machinery. Nuclear export of microRNAs (miRNAs) by exportin-5 (Exp-5) is an essential step in miRNA biogenesis. In addition, Exp-5 protects pre-miRNAs from digestion by nucleases [23]. The Exp-5:RanGTP:pre-miRNA heteroternary complex formed in the nucleus is exported to the cytoplasm. Ran GTPase–activating protein, which causes GTP hydrolysis together with RanBP1 and/or RanBP2, is localized in the cytoplasm and induces the conformational change of Ran to release the pre-miRNA cargo from Exp-5 [23].

Domains 1 of *C. intestinalis* and *B. floridae* Htts have shown structural similarity with Serine/threonine-protein phosphatase 2A 56 kDa regulatory subunit (PDB codes: 2IAE, 3FGA, respectively [24,28]).

Protein phosphatase 2A (PP2A) regulates many aspects of cellular activities [24]. For example, it regulates DNA replication, transcription, translation, cell cycle, development, and apoptosis. To execute these functions, it is subjected to specificity control through the formation of hetero-oligomers, where the catalytic subunits are complexed with regulatory subunits [29]. Indeed, PP2A is a holoenzyme and the core enzyme is made up of a 65-kDa scaffolding A subunit and a 36-kDa catalytic C subunit. The binding of one of at least 18 regulatory B subunits to the AC core enzyme regulates PP2A activities. B subunits have been implicated in controlling PP2A substrate specificity, cellular localization, and enzymatic activity. On the basis of sequence homology, regulatory B subunits can be classified into B (B55), B9 (B56) and B99 families [24].

There is no evidence of HsHtt function in phosphorylation; however, given the conservation of some residues involved in the interaction with A and C subunits of the protein phosphatase 2A, domain 1 could have a role in the phosphorylation of other molecules. Furthermore, three ordered domains of HsHtt have shown structural similarity with subunit A of protein phosphatase 2A [17].

The structural models of the domains 2 of *C. intestinalis* and *B. floridae* display structural similarity with Importin β (PDB code: 1IBR [25]). Karyopherin β/Importin β (Impβ) is the most important mediator of nuclear import of proteins that carry a classical nuclear localization sequence (NLS), through an adaptor, Importin α. RanGTP displaces Impα, directly binding import substrates setting free Impβ from certain sites of the NPCs [25].

Domain 3 and 4 of *C. intestinalis* and domain 3 of *B. floridae* Htt have shown a fold similar to that of the Importin 13 (PDB codes: 2X1G for domain 3 of *C. intestinalis* and 2XWU for domain 4 of *C. intestinalis* and domain 3 of *B. floridae* [26,27]. Karyopherins can be divided into two groups on the basis of their directionality: importins transport cargoes from the cytoplasm to the nucleus, while exportins from the nucleus into the cytoplasm. Only a few karyopherins, including Importin13 (Imp13), can mediate both import and export of molecules. However, it is still unclear how Importin 13 can have a double transport ability. RanGTP binding by Imp13 is similar to RanGTP binding by Impβ even if Imp13 lacks the acidic loop that is found in the canonical import factors [31].

In human cells, Imp13 can export eIF1A, the translation initiation factor and import the exon junction complex components Mago-Y14 as well as several transcription factors and the E2 SUMO-conjugating enzyme Ubc9. Mago-Y14 is the most studied example of Imp13-mediated transport, whereas how Imp13 mediates the nuclear import of Ubc9 is not known [31]. Interestingly, in [32], models of a ternary complex between RHES (“Ras homolog enriched in striatum”, a GTP binding protein with similarity to Ras family members), the third ordered domain of HsHtt and SUMO-E2 ligase Ubc9 have been built by molecular docking [32]. This could be useful to understand the mechanism of transport of Ubc9 regulated by Imp13.

Domain 4 of *B. floridae* and domain 2 of *C. elegans* display a fold similar to that of the exportin Xpo4 (PDB code: 5DLQ [22]). Xpo4 is a bidirectional nuclear transport receptor that mediates nuclear export of eIF5A (Eukaryotic Translation Initiation Factor 5A) and Smad3 (small mother against decapentaplegic) as well as import of Sox-type transcription factors and possibly other proteins into the nucleus [22]. How Xpo4 can recognize such a variety of cargoes is still unclear. The recognition mechanism of Ran by Xpo4 is similar to that observed in other exportins and, also in this case, RanGTP increases the affinity for the cargoes.

The first domain of *C. elegans* has shown structural similarity with the Karyopherin Kap121p (PDB code: 3W3T [30], the same structural homolog identified for the third ordered domain of HsHtt [17]. Usually, Karyopherins are responsible for importing cargoes and are called importins while exporters are called exportins. The direction of transport for each karyopherin depends on its differential interactions with cargoes and the small GTPase Ran [30]. Importins bind their cargoes in the cytoplasm and release cargoes in the nucleus upon RanGTP binding.

## 4. Materials and Methods

The sequences of the amoeba, the ascidian, and the amphioxus Htts, and of the Htt-like protein identified in *C. elegans* were retrieved through a delta-BLAST (Domain Enhanced Look-up Time Accelerated BLAST) [33] search using HsHtt sequence as a bait against the non-redundant protein sequences database. Delta-BLAST aligns a query sequence to conserved domains in CDD (Conserved Domain Database) [34] through RPS-BLAST (which stands for “Reverse Position-Specific BLAST and is a variant of the popular PSI-BLAST program, “Position-Specific Iterated BLAST”) and then performs a sequence database search using a PSSM (position specific scoring matrix) [35,36,37] derived from the aligned domains. A PSSM is obtained from a multiple sequence alignment (MSA) of related proteins. Delta-BLAST uses aligned domains to compute a PSSM and to find more homologs [33].

The multiple sequence alignment between *H. sapiens*, *C. intestinalis*, *B. floridae*, *D. discoideum* Htt, and *C. elegans* Htt-like proteins has been performed using Clustal Omega [38] and visualized with Jalview [39]. In Clustal Omega, the alignments are computed using the very accurate HHalign package [40], which aligns two hidden Markov model profiles [41].

Disordered regions and ordered domains have been identified by several tools, described below.

Foldindex (https://fold.weizmann.ac.il/fldbin/findex accessed on 1 June 2020) predicts if a protein sequence is intrinsically disordered [42]. Its algorithm takes into account the average hydrophobicity and the net charge of the sequence and calculates a ‘foldability’ score [43].

Globplot (http://globplot.embl.de/cgiDict.py last accessed on 1 June 2020) detects the disordered regions of a protein by measuring the order/disorder propensity of sequence segments [44].

PredictProtein (https://www.predictprotein.org last accessed on 1 June 2020) is a sequence-based meta-service that predicts a variety of structural and functional properties of proteins, providing also a reliability score to judge the quality of the prediction [45].

ANCHOR (https://anchor.enzim.hu/ last accessed on 1 June 2020) is a web service used to identify protein regions that become ordered only after binding to a molecular partner [46]. ANCHOR uses energy calculations to infer the biophysical properties of disordered binding regions [47]. ANCHOR’s score corresponds to the probability that a residue is part of a disordered binding region. Regions with an overall score >0.5 are predicted to be disordered binding regions [46]. Currently ANCHOR is part of the IUPred2A web service (https://iupred2a.elte.hu/ last accessed on 1 June 2020).

In the InterPro database (https://www.ebi.ac.uk/interpro/ last accessed on 1 June 2020), sequences are classified into protein families. This classification, through signatures diagnostic models, is used to predict the presence of functional domains and sites [48,49,50].

SMART (http://smart.embl-heidelberg.de/ last accessed on 1 June 2020) is a web resource for the identification of protein domains and the analysis of their architectures; it is synchronized with UniProt, ENSEMBL [51] and STRING [52]. The SMART database integrates manually-curated hidden Markov models for many domains [53] and offers a variety of analysis and visualization tools [54,55].

HMMER (http://www.ebi.ac.uk/Tools/hmmer/ last accessed on 1 June 2020) is a suite for sequence similarity searches using profile hidden Markov models (HMMs) [56] focused primarily on UniProtKB. The query sequence is scanned against the Pfam profile HMM library using hmmscan to detect any Pfam family [57] and analyzed for the presence of disordered regions, using IUPred [58,59], signal peptides and transmembrane regions, using Phobius [60], and coiled-coil regions [61].

MobiDB 3.0 (http://mobidb.bio.unipd.it/ last accessed on 1 June 2020), [62] is an update of the previous database of intrinsically disordered and mobile proteins, MobiDB 2.0 [63], which provides a complete picture of different types of protein disorder covering all Uniprot sequences.

MobiDB 3.0 returns a consensus of different predictors: ESpritz [64], IUPred [58], DisEMBL [65], GlobPlot [44], VSL2b [66], DynaMine [67], Anchor [47], FeSS [68]. Consensus generation is handled by MobiDB-lite [69].

PONDR (http://www.pondr.com last accessed on 1 June 2020) predicts short, medium, and long disordered regions using different neural network-based predictors. In this work, PONDR VL-XT was used, which integrates the VL1 predictor (for internal regions), the N-terminus predictor (XN), and the C-terminus predictor (XC) [70].

SPOT-DISORDER (http://sparks-lab.org/server/SPOT-disorder/ last accessed on 1 June 2020) implements a deep bidirectional LSTM (Long Short-Term Memory) recurrent neural networks to capture nonlocal interactions that are essential for determining the structured or intrinsically disordered nature of a protein sequence [71].

Tables S2, S8, S14 and S20 display the ordered and disordered regions identified in *D. discoideum*, *C. intestinalis*, *B. floridae* and *C. elegans* protein sequences, respectively.

The I-TASSER server (https://zhanglab.ccmb.med.umich.edu/I-TASSER/ last accessed on 1 June 2020) has been used to carry out the structure prediction of protein domains. I-TASSER is an integrated platform to perform protein structure and function prediction. It is based on a threading approach to identifying suitable modelling templates for the query sequence [72]. The quality of the template alignments is evaluated through a Z-score that corresponds to the statistical significance of the best threading alignment [72]. Models are built using a combination of threading and ab initio techniques for the regions aligned to the template and those that are not, respectively. The function of the input protein is also predicted by structural homology of the models with known protein structures [72]. Tables S4–S7, S10–13, S16–19, S22 and S23 show the structural homologs of the domains of the Htt-like proteins of *D. discoideum*, *C. intestinalis* and *B. floridae* and in *C. elegans*, respectively. The reliability of the structural models can be assessed by the C-score parameter provided by the server. Table S3, S9, S15 and S21 report the C-score values obtained for the models of the predicted ordered domains of Htt sequences of *D. Discoideum*, *C. intestinalis*, *B. floridae*, and of the Htt-like sequence of *C. elegans*, respectively.

## 5. Conclusions

In conclusion, in the present work, a computational analysis of Htt sequences of selected model organisms was performed with the aim to understand the evolution of the structure and function of huntingtin. In *Dictyostelium discoideum* amoeba, the first organism shown to carry an Htt gene, 19 CAG triplets are located in a different position with respect to the human counterpart, suggesting that in the deuterostomes branch, there was first a decrease of CAG triplets with a subsequent increase in primates and even more in *Homo sapiens*. A Htt-like protein, not previously identified, has been detected for the first time in *C. elegans*. Four ordered domains, instead of the five characterizing the human protein, have been identified in *D. discoideum*, *C. intestinalis*, and *B. floridae* Htts, in agreement with the shorter length of the sequences with respect to human protein one. In *C. elegans* sequence, which is much shorter than all of the above, only two ordered domains have been detected. This “minimal” Htt-like protein seems to indicate that while the intracellular trafficking function of Htt is common to all of the organisms analysed, the specific functions that Htt exerts in the nervous system require the presence of other domains.

The analyses of Htt sequences of *D. discoideum*, *C. intestinalis*, *B. floridae,* and *C. elegans* have shown the conservation of the third ordered human domain in these organisms, less evident in the amoeba. However, this domain could have a similar function in vesicular transport, but not necessarily in neuronal tissues. On the contrary, the other domains of HsHtt seem to be conserved only in the proteins from *C. intestinalis* and *B. floridae*, which display a length comparable to that of the human protein. The lack of two portions in the nematode Htt-like protein suggests that the ancestral Htt sequence could be similar to that of the nematode one, in terms of ordered domains, and that the proteins found in higher complexity organisms have acquired additional domains and, consequently, additional functions.

## Figures and Tables

**Table 1:** HEAT repeats location within HsHtt [1], CiHtt, and amphioxus Htt (BfHtt) [15].

HsHtt	CiHtt	BfHtt
N-terminal 114–413	N-terminal 58–96	N-terminal 75–113
N-terminal 672–1176	N-terminal 139–177	N-terminal 156–194
Central 1289–1710	N-terminal 181–219	N-terminal 198–236
Central 2175–2325	N-terminal 682–720	N-terminal 306–344
C-terminal 2355–2475	Central 867–905	N-terminal 802–840
C-terminal 2667–2937	Central 1341–1378	Central 1371–1409
C-terminal 2975–3107	C-terminal 2771–2809	Central 1556–1595
	C-terminal 2864–2904	Central 1618–1656
		C-terminal 2746–2784
		C-terminal 2927–2965
		C-terminal 3020–3038

**Table 2:** Summary of the structural homologues analyzed for each ordered domain of the Htt proteins.

Organism	Domain	Structural Homologue	RMSD (Å)	Coverage (%)	Sequence Identity (%)
*Dictyostelium discoideum*	1	DCB-HUS domain of *Thermothielavioides terrestris* Sec7 (PDB: 5HAS [19])	2.23	94.4	9.8
	2	exportin Cse1 (PDB: 1Z3H [21])	6.07	61.3	7.8
	3	Exportin (PDB: 5DLQ [22])	5.40	70.1	8.3
	4	Exportin-5 (Exp-5) (PDB: 3A6P [23])	6.72	62.1	8.1
*Ciona intestinalis*	1	serine/threonine-protein phosphatase 2A (PDB: 2IAE [24])	4.27	91.0	8.8
	2	Importin β (Impβ) (PDB: 1IBR [25])	4.89	63.8	8.4
	3	Importin 13 (PDB: 2X1G [26])	6.33	44.9	6.3
	4	Importin 13 (PDB: 2XWU [27])	5.44	86.1	5.9
*Branchiostoma floridae*	1	Serine/threonine-protein phosphatase 2A (PDB: 3FGA [28]	4.26	86.3	11.0
	2	Importin β (PDB: 1IBR [25])	5.52	74.4	10.1
	3	Importin13 (PDB: 2XWU [27])	7.04	66.0	7.7
	4	Exportin Xpo4 in complex con RanGTP (PDB: 5DLQ [22])	7.53	68.5	8.0
*Caenorhabditis elegans*	1	Karyopherin Kap121p (PDB: 3W3T [30])	6.86	63.7	7.5
	2	Exportin Xpo4 in complex with RanGTP (PDB: 5DLQ [22])	7.40	77.2	6.1

## Data Availability

Original data are available upon request to the corresponding author.

## Supplementary Materials

Supplementary materials can be found at https://www.mdpi.com/1422-0067/22/6/3214/s1.

## Abbreviations

HD	Huntington’s disease
Htt	huntingtin
polyQ	Polyglutamine
HsHtt	*Homo sapiens* Htt
DdHtt	*Dictyostelium discoideum* Htt
CiHtt	*Ciona intestinalis* Htt
BfHtt	*Branchiostoma floridae* Htt
