# Supplementary material for: In Silico Analysis of Huntingtin Homologs in Lower Eukaryotes
Source: Int J Mol Sci. 2021 Mar 22;22(6):3214. doi: 10.3390/ijms22063214 (PMC8004120; doi:10.3390/ijms22063214)
Supplement: Supplementary file 1 [file ijms-22-03214-s001.pdf]

In silico analysis of huntingtin homologs in lower eukaryotes. Identification of a huntingtin-like protein in Caenorhabditis elegans.

Valentina Brandi[1] and Fabio Polticelli [1, 2*]

[1] Department of Sciences, Roma Tre University, 00146 Rome, Italy

[2] National Institute of Nuclear Physics, Roma Tre University, 00146 Rome, Italy

* Correspondence: Prof. Fabio Polticelli; fabio.polticelli@uniroma3.it; Tel.: +39-06-5733-6362

Supplementary materials

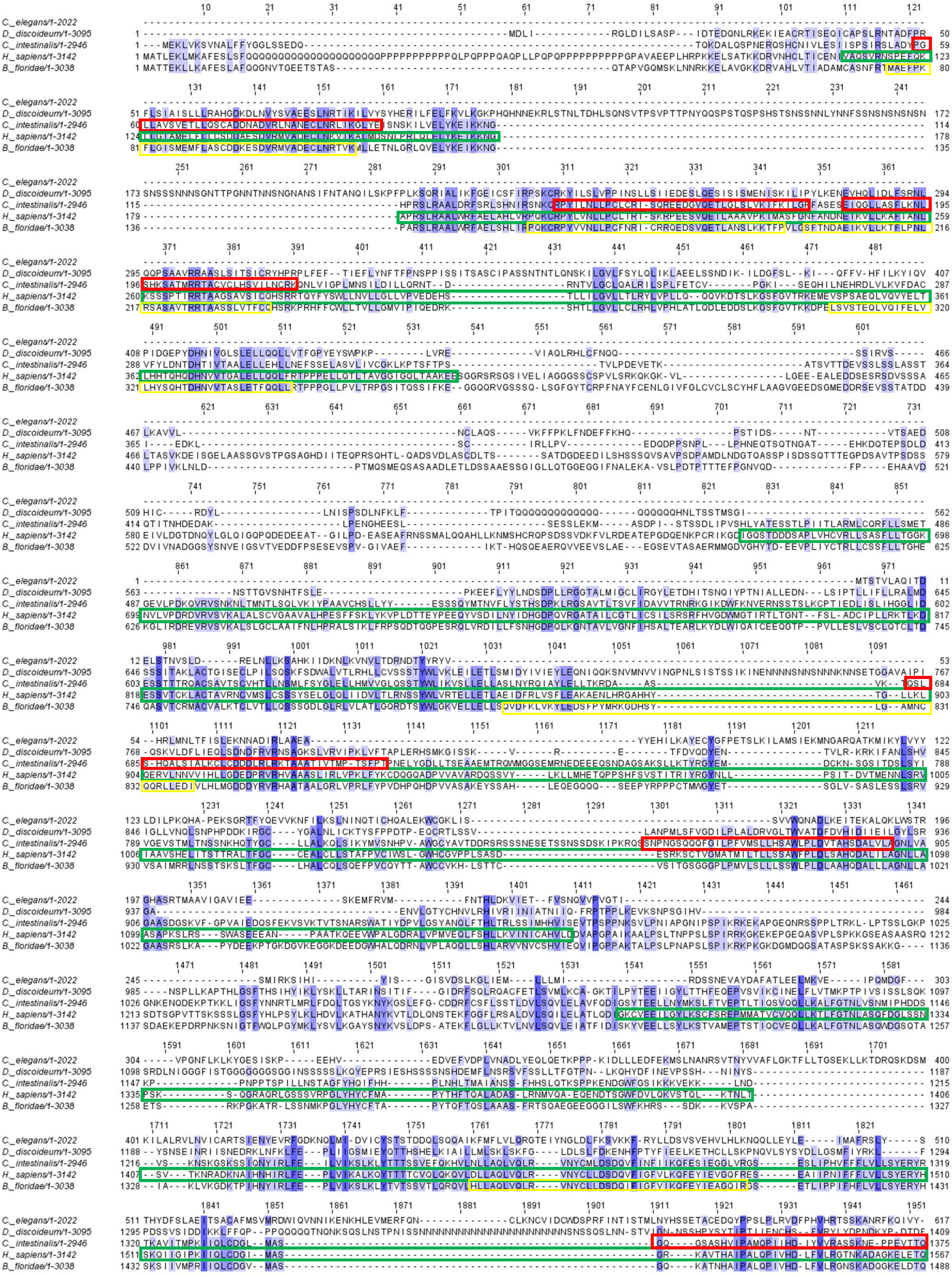
C._elegans/1-2022
D._discoideum/1-3095
C._intestinalis/1-2946
H._sapiens/1-3142
B._floridae/1-3038

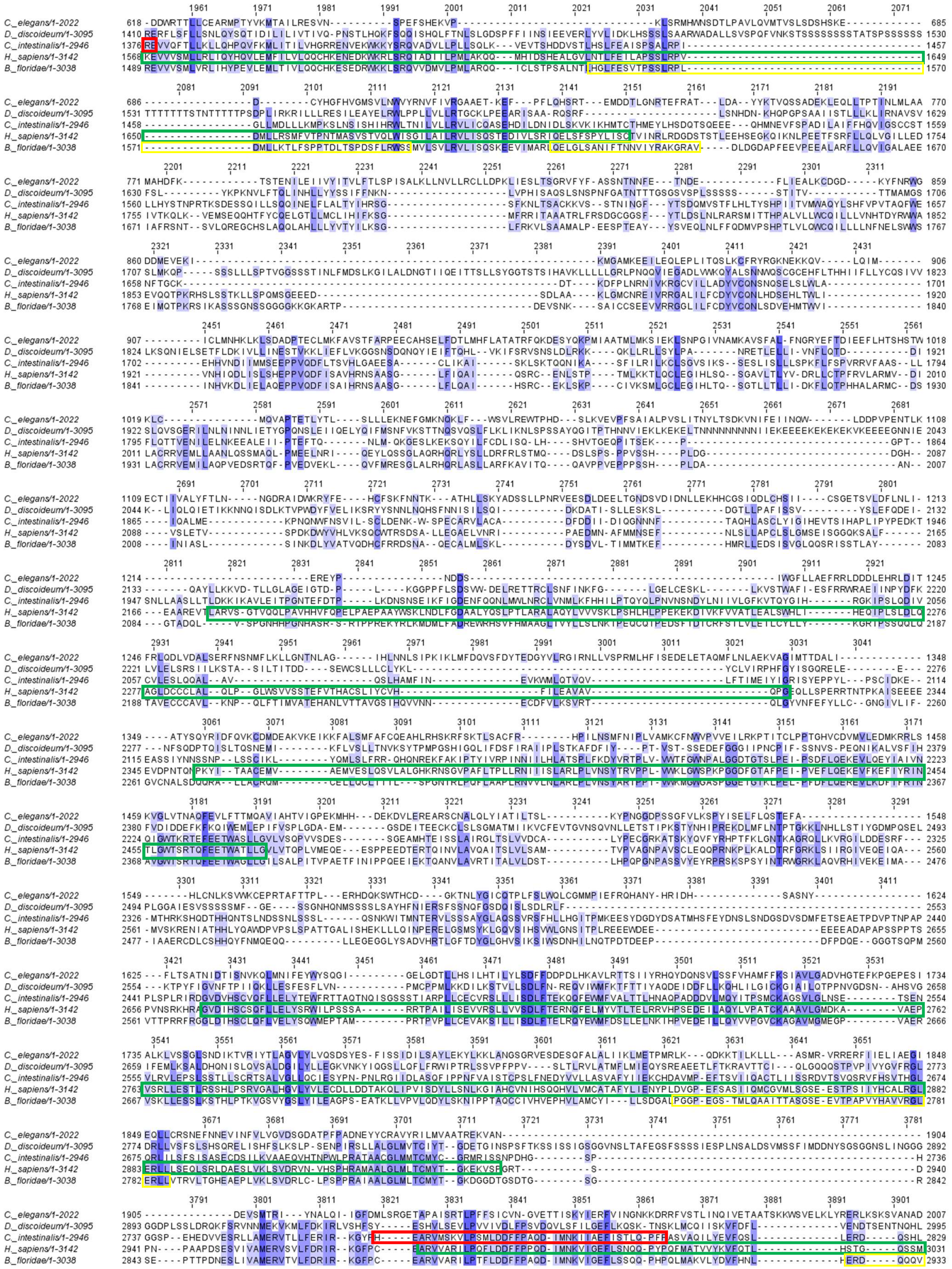
C_elegans/1-2022
D_discoideum/1-3095
C_intestinalis/1-2946
H_sapiens/1-3142
B_floridae/1-3038

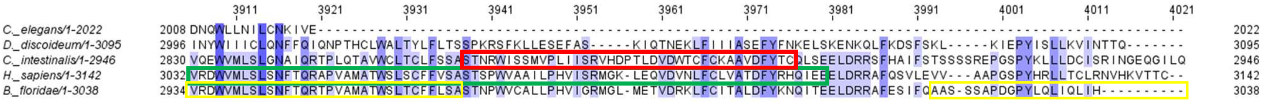

Figure S1. Multiple sequence alignment between *H. sapiens*, *C. intestinalis*, *B. floridae, D. discoideum* Htt and *C. elegans* Htt-like. Residues are coloured by percentage identity. The red, green and yellow boxes indicate HEAT repeats in ascidian, in human and amphioxus Htt, respectively.

Table S1. Query coverage, *E*-value and percentage identity between human, ascidian, amphioxus, amoeba Htt sequences and *C. elegans* Htt-like sequence, obtained through distinct BLAST searches.

Blast hit	Query coverage	*E*-value	Identity	Accession number
Ciona intestinalis **Htt**	88%	0.0	34%	CAJ87484.4
Branchiostoma floridae **Htt**	99%	0.0	46%	CAM12495.1
Dictyostelium discoideum **Htt**	33%	2x10^{-19}	29%	XP_645159.1
Caenorhabditis elegans	13%	3x10^{-6}	21%	NP509663.3
C. elegans domain 1	21%	8x10^{-4}	38%	
C. elegans domain 2	49%	5x10^{-9}	21%	

Table S2. Disordered regions identified in *D. discoideum* Htt sequence.

Method	Disordered regions
**Foldindex (Uversky et al., 2000)**	109-203, 510-572, 727-742, 756-768, 1302-1390, 1985-2061
**Predictprotein (Yachdav et al., 2014)**	530-551, 970-980, 1316-1326, 1511-1543, 2004-2041
**SMART (Letunic and Bork, 2018)**	Low complexity regions: 140-199, 1103-1127, 1135-1145, 1180-1200, 1305-1368, 1425-1438, 1510-1547, 1568-1579, 1679-1701, 1752-1764, 1885-1901, 1930-1939, 2000-2054, 2249-2259, 2501-2525, 2833-2842, 2852-2864, 2878-2900
**HMMER (Potter et al, 2018)**	109-204, 539-558, 740-757, 1123-1133, 1306-1370, 1515-1541, 1995-2038
**MOBIDB (Piovesan et al., 2018)**	105-197, 536-561, 1312-1371, 1509-1547, 2005-2037
**GLOBPLOT (Linding, Russell, et al., 2003)**	1-12, 174-251, 383-407, 609-623, 787-812, 927-938, 1022-1045, 1151-1179, 1188-1199, 1366-1433, 1559-1599, 1717-1745, 1765-1781, 2200-2216, 2395-2416, 2539-2550, 2554-2573, 2872-2901, 2926-2953<br>DOMAIN: 2-173, 252-382, 408-608, 624-790, 813-1021, 1046-1150, 1180-1373, 1434-1558, 1600-1716, 1782-2199, 2217-2394, 2417-2553, 2574-2871, 2954-3147

**SPOT-DISORDER (Hanson et al., 2017)**	1-20, 125-192, 492-571, 735-760, 971-988, 1099-1130, 1317-1364, 1511-1546, 1670-1713
**PONDR VLXT (Romero et al., 2001)**	13-36, 119-197, 249-266, 342-356, 537-568, 721-761, 979-987, 1111-1146, 1185-1198, 1319-1380, 1507-1548, 1668-1723, 2272-2285, 2401-2419, 2488-2520, 2731-2746, 2850-2869, 2882-2895, 2903-2913
**Iupred2A (Mészáros et al., 2018)**	Domains: 1-101, 207-1304, 1375-1993, 2045-3095

Table S3. C-Score of the models of *D. discoideum* Htt ordered domains, obtained with I-TASSER.

Ordered domains of *Dictyostelium* Htt	C-score
**Domain 1**	0.20
**Domain 2**	-1.19
**Domain 3**	-2.10
**Domain 4**	-0.35

Table S4. Structural homologs of domain 1 of *D. discoideum*, identified by I-TASSER.

Rank	PDB hit	TM-score	RMSD (Å)	Identity (%)	Coverage
**1**	6EZ8	0.776	0.76	0.368	0.806
**2**	5HAS	0.759	2.23	0.098	0.944
**3**	3L5Q	0.730	2.44	0.050	0.935
**4**	5VCH	0.730	2.43	0.050	0.935
**5**	3W3T	0.720	2.47	0.113	0.898
**6**	3S4W	0.718	2.94	0.085	0.972
**7**	6COK	0.716	2.24	0.092	0.907
**8**	2IWH	0.715	2.54	0.150	0.889
**9**	3FGA	0.713	2.51	0.071	0.907
**10**	4ATG	0.711	2.36	0.122	0.907

Table S5. Structural homologs of domain 2 of *D. discoideum*, identified by I-TASSER.

Rank	PDB hit	TM-score	RMSD (Å)	Identity (%)	Coverage
**1**	6EZ8	0.771	1.18	0.233	0.778
**2**	1Z3H	0.492	6.07	0.078	0.613
**3**	2BKU	0.475	5.56	0.066	0.572
**4**	1QGR	0.474	6.23	0.086	0.602
**5**	6EMK	0.470	6.75	0.080	0.618

**6**	2X1G	0.468	7.20	0.061	0.627
**7**	4XRI	0.463	5.84	0.088	0.568
**8**	5GM6	0.459	5.87	0.075	0.563
**9**	5HB4	0.457	7.52	0.044	0.620
**10**	5DLQ	0.457	7.95	0.083	0.650

Table S6. Structural homologs of domain 3 of *D. discoideum*, identified by I-TASSER.

Rank	PDB hit	TM-score	RMSD (Å)	Identity (%)	Coverage
**1**	6EZ8	0.765	1.57	0.170	0.783
**2**	5HB4	0.565	5.67	0.073	0.754
**3**	5NVR	0.549	5.65	0.081	0.731
**4**	5DLQ	0.543	5.40	0.083	0.701
**5**	5T8V	0.529	6.91	0.067	0.773
**6**	3A6P	0.525	6.67	0.065	0.769
**7**	6REY	0.520	6.23	0.087	0.718
**8**	2JKR	0.517	5.64	0.102	0.694
**9**	6N1Z	0.517	5.69	0.050	0.681
**10**	6H7W	0.506	5.96	0.068	0.688

Table S7. Structural homologs of domain 4 of *D. discoideum*, identified by I-TASSER.

Rank	PDB hit	TM-score	RMSD (Å)	Identity (%)	Coverage
**1**	6EZ8	0.812	2.01	0.207	0.834
**2**	3A6P	0.480	6.72	0.081	0.621
**3**	5DLQ	0.479	8.13	0.061	0.686
**4**	3M1I	0.469	7.73	0.064	0.654
**5**	5HB4	0.457	6.71	0.069	0.592
**6**	4KF7	0.455	7.66	0.063	0.627
**7**	5IJO	0.444	6.90	0.070	0.584
**8**	3GB8	0.441	7.27	0.061	0.592
**9**	5GM6	0.435	6.45	0.049	0.555
**10**	4FGV	0.434	7.24	0.050	0.578

Table S8. Disordered regions identified in *C. intestinalis* Htt sequence.

Method	Disordered regions
**Foldindex (Uversky et al., 2000)**	102-139, 370-445, 726-776, 833-863, 981-1056, 1182-1231, 1510-1523, 1546-1550, 1867-1871, 2121-2130, 2133-2138, 2209-2213, 2298-2356, 2383-2387, 2389-2436

**Predictprotein (Yachdav et al., 2014)**	18-29, 336-356, 373-449, 745-758, 835-865, 974-1036, 1204-1222, 1853-1863, 2408-2438, 2729-2743
**Anchor (Dosztányi et al., 2009)**	357-378, 411-420, 434-443, 452-461, 470-481, 951-969, 1040-1051, 2366-2387
**SMART (Letunic and Bork, 2018)**	303-317, 355-363, 431-440, 693-705, 834-854, 896-908, 1760-1784, 1946-1957, 2341-2355, 2562-2575
**HMMER (Potter et al, 2018)**	339-354, 375-444, 835-840, 842-861, 981-1027, 2328-2352, 2415-2442
**MOBIDB (Piovesan et al., 2018)**	380-447, 836-866, 876-1037, 2329-2350, 2420-2444
**GLOBPLOT (Linding, Russell, et al., 2003)**	404-456, 742-751, 863-897, 999-1061, 1165-1185, 1215-1229, 1880-1891, 1998-2011, 2215-2231, 2362-2377, 2418-2474, 2751-2769, 2944-2954; Globular domains: 2-403, 442-862, 898-998, 1062-1164, 1230-1997, 2012-2214, 2232-2361, 2475-2750, 2770-2950
**SPOT-DISORDER (Hanson et al., 2017)**	1-30, 330-452, 746-760, 836-867, 923-935, 976-1036, 1142-1157, 1209-1222, 1853-1864, 2391-2440, 2726-2745
**INTERPRO (Finn et al., 2017)**	23-324, 471-988, 2334-2915
**PONDR VLXT (Romero et al., 2001)**	25-43, 50-60, 112-132, 154-164, 305-313, 337-363, 369-463, 567-576, 719-759, 838-863, 968-1024, 1142-1157, 1206-122, 1338-1351, 1358-1377, 1405-1415, 1524-1539, 1567-1579, 1706-1725, 1802-1820, 1851-1864, 1972-1981, 2243-2266, 2310-2324, 2340-2352, 2414-2446, 2547-2562, 2727-2753

Table S9. C-Score of the models of ascidian Htt ordered domains, obtained with I-TASSER. The models have been selected by Confidence-score, a parameter to evaluate the quality of predicted models. Generally, C-score can range from -5 to 2, where a higher value corresponds to a greater level of confidence of the model.

Ordered domains of ascidian Htt	C-score
**Domain 1**	-0.92
**Domain 2**	-0.28
**Domain 3**	0.59

Domain 4	0.33

Table S10. Structural homologs of domain 1 of ascidian Htt, identified by I-TASSER. TM-score and RMSD are known standards for measuring structural similarity between two structures which are usually used to measure the accuracy of structure modelling when the native structure is known.

Rank	PDB hit	TM-score	RMSD (Å)	Identity (%)	Coverage
1	6EZ8	0.857	0.86	0.397	0.870
2	5YZ0	0.734	4.18	0.071	0.948
3	4A0C	0.700	3.43	0.101	0.849
4	5VCH	0.697	4.19	0.108	0.910
5	5IFE	0.694	4.13	0.079	0.901
6	1VSY	0.691	4.39	0.091	0.914
7	2IAE	0.687	4.26	0.088	0.910
8	3W3T	0.686	4.19	0.103	0.901
9	2JAK	0.685	4.42	0.077	0.910
10	5DLQ	0.682	4.81	0.112	0.957

Table S11. Structural homologs of domain 2 of ascidian Htt, identified by I-TASSER.

Rank	PDB hit	TM-score	RMSD (Å)	Identity (%)	Coverage
1	6EZ8	0.828	1.13	0.288	0.840
2	5DLQ	0.525	6.19	0.085	0.744
3	4UVK	0.518	6.04	0.103	0.717
4	1VSY	0.515	5.72	0.079	0.696
5	2X1G	0.508	5.54	0.076	0.686
6	3ICQ	0.503	6.07	0.083	0.702
7	1IBR	0.503	4.89	0.084	0.638
8	1U6G	0.499	5.75	0.102	0.683
9	2XWU	0.499	6.40	0.067	0.721
10	4KNH	0.498	6.28	0.083	0.719

Table S12. Structural homologs of domain 3 of ascidian Htt, identified by I-TASSER.

Rank	PDB hit	TM-score	RMSD (Å)	Identity (%)	Coverage
1	6EZ8	0.866	0.81	0.354	0.870
2	5T8V	0.389	8.26	0.060	0.541
3	5HB4	0.372	8.11	0.044	0.516
4	6BQ1	0.365	8.96	0.037	0.531
5	2X1G	0.362	6.33	0.063	0.449
6	2XWU	0.357	7.15	0.057	0.463

**7**	1VSY	0.356	7.60	0.065	0.478
**8**	4C0O	0.354	6.86	0.061	0.455
**9**	3ICQ	0.352	7.15	0.052	0.460
**10**	1WA5	0.351	6.55	0.044	0.441

Table S13. Structural homologs of domain 4 of ascidian Htt, identified by I-TASSER.

Rank	PDB hit	TM-score	RMSD (Å)	Identity (%)	Coverage
**1**	6EZ8	0.870	1.07	0.458	0.882
**2**	3ZKV	0.651	5.23	0.061	0.863
**3**	2XWU	0.638	5.44	0.059	0.861
**4**	4C0O	0.632	5.10	0.088	0.846
**5**	1W63	0.630	4.40	0.075	0.784
**6**	3DW8	0.628	4.67	0.084	0.803
**7**	3NBZ	0.627	5.48	0.078	0.874
**8**	3ICQ	0.619	5.30	0.071	0.833
**9**	1U6G	0.618	4.68	0.078	0.791
**10**	5DLQ	0.618	5.72	0.071	0.863

Table S14. Disordered regions identified in *B. floridae* Htt sequence

Method	Disordered regions
**Foldindex (Uversky et al., 2000)**	27-39, 430-452, 517-532, 545-575, 577-592, 868-908, 1025-1059, 1108-1162, 1260-1285, 1777-1798, 2083-2127, 2441-2472, 2515-2569
**Predictprotein (Yachdav et al., 2014)**	19-50, 416-439, 447-479, 490-532, 534-592, 1030-1051, 1088-1147, 1254-1269, 1780-1799, 2537-2560, 2831-2848
**Anchor (Dosztányi et al., 2009)**	7-16, 474-496, 514-539, 1058-1088, 1097-1107
**SMART (Letunic and Bork, 2018)**	217-230, 268-278, 476-488, 984-1024, 1039-1053, 1096-1108, 1123-1141, 1365-1381, 1686-1697, 1747-1758, 1774-1794, 1991-2002, 2494-2505, 2538-2552, 2827-2843
**HMMER (Potter et al, 2018)**	27-43, 426-450, 493-520, 883-892, 1031-1056, 1095-1146, 1782-1791, 2091-2103, 2537-2560, 2831-2849
**MOBIDB**	422-441, 1031-1051, 1091-1150, 1777-1798, 2536-2561

**(Piovesan et al., 2018)**	
**GLOBPLOT (Linding, Russell, et al., 2003)**	373-385, 402-416, 450-474, 505-514, 528-542, 714-722, 927-941, 1008-1021, 1060-1080, 1118-1182, 1284-1301, 1606-1618, 1806-1827, 2020-2034, 2116-2135, 2357-2375, 2459-2483, 2565-2595, 2771-2781, 2854-2878 Globular domain: 1-401, 543-926, 1183-1805, 1828-2019, 2035-2115, 2136-2356, 2376-2458, 2477-2564, 2596-2853, 2879-3038
**SPOT-DISORDER (Hanson et al., 2017)**	1-50, 370-594, 1033-1050, 1092-1148, 1254-1268, 1298-1308, 1778-1805, 1990-2006, 2538-2564, 2831-2848
**PONDR (Romero et al., 2001)**	21-56, 128-139, 349-357, 419-482, 491-516, 527-600, 880-935, 981-995, 1024-1034, 1089-1147, 1256-1271, 1322-1331, 1479-1498, 1520-1533, 1607-1619, 1642-1662, 1773-1809, 1944-1963, 1990-2008, 2071-2120, 2320-2334, 2398-2417, 2428-2464, 2537-2557, 2658-2670, 2742-2776, 2788-2805, 2832-2855

Table S15. C-Score of the models of AmphiHtt ordered domains obtained with I-TASSER

Ordered domains of AmphiHtt	C-score
**Domain 1**	0.04
**Domain 2**	-0.67
**Domain 3**	0.50
**Domain 4**	0.49

Table S16. Structural homologs of domain 1 model of AmpiHtt, identified by I-TASSER

Rank	PDB hit	TM-score	RMSD (Å)	Identity (%)	Coverage
**1**	6EZ8	0.897	1.32	0.589	0.916
**2**	4A0C	0.679	3.46	0.091	0.826
**3**	2BKU	0.677	3.86	0.102	0.845
**4**	1VSY	0.661	4.46	0.089	0.867
**5**	4XRI	0.661	3.82	0.068	0.829
**6**	3FGA	0.656	4.26	0.110	0.863
**7**	5IFE	0.649	4.27	0.087	0.845
**8**	3S4Z	0.642	4.34	0.089	0.867
**9**	5DLQ	0.638	4.76	0.090	0.891

Table S17. Structural homologs of domain 2 model of AmpiHtt, identified by I-TASSER

Rank	PDB hit	TM-score	RMSD (Å)	Identity (%)	Coverage
1	6EZ8	0.890	2.74	0.366	0.956
2	4KF7	0.579	5.10	0.071	0.774
3	4KNH	0.542	5.54	0.080	0.755
4	1IBR	0.540	5.52	0.101	0.744
5	3NBY	0.538	5.16	0.071	0.737
6	4C0O	0.538	5.26	0.107	0.730
7	5VCH	0.538	5.54	0.077	0.741
8	3ND2	0.538	5.63	0.092	0.741
9	4XRI	0.537	4.82	0.059	0.693
10	1VSY	0.535	6.08	0.062	0.797

Table S18. Structural homologs of domain 3 model of AmpiHtt, identified by I-TASSER

Rank	PDB hit	TM-score	RMSD (Å)	Identity (%)	Coverage
1	6EZ8	0.824	0.89	0.562	0.829
2	3ICQ	0.495	6.38	0.070	0.642
3	2XWU	0.482	7.04	0.077	0.660
4	6EMK	0.474	7.17	0.054	0.648
5	4C0O	0.472	6.74	0.047	0.633
6	3EA5	0.470	6.76	0.067	0.630
7	5HB4	0.469	7.75	0.071	0.673
8	6BCU	0.461	6.55	0.088	0.607
9	4A0C	0.461	6.45	0.065	0.604
10	5DLQ	0.461	6.65	0.052	0.610

Table S19. Structural homologs of domain 4 model of AmpiHtt identified by I-TASSER

Rank	PDB hit	TM-score	RMSD (Å)	Identity (%)	Coverage
1	6EZ8	0.839	0.61	0.528	0.842
2	5HB4	0.503	6.50	0.061	0.663
3	4KF7	0.502	6.22	0.085	0.644
4	5IJO	0.488	6.20	0.068	0.628
5	5DLQ	0.480	7.53	0.080	0.685
6	5YZ0	0.479	6.59	0.055	0.632
7	5GM6	0.477	6.34	0.065	0.616
8	3M1I	0.473	6.60	0.076	0.625
9	3NBY	0.473	6.51	0.062	0.620

**10**	3W3T	0.472	5.89	0.075	0.586

Table S20. Disordered regions identified in the Htt-like sequence of *C. elegans*

Method	Disordered regions
**Foldindex (Uversky et al., 2000)**	299-303, 317-347, 561-566, 594-598, 728-741, 835-864, 871-880, 1142-1199, 1224-1228, 1231-1240, 1971-1989
**Predictprotein (Yachdav et al., 2014)**	1, 129, 314-325, 340-348, 1161-1164, 1488-1489, 1494
**SMART (Letunic and Bork, 2018)**	801-812, 1027-1041, 1232-1243
**MOBIDB (Piovesan et al., 2018)**	1-5, 646-651, 653-654, 731, 1168-1169, 1495-1496, 1793-1794
**GLOBPLOT (Linding, Russell, et al., 2003)**	630-644, 729-733, 1482-1487, 1564-1578, 1621-1632, 1769-1774, 1917-1926<br>DOMAINS: 3-629, 645-1563, 1579-2068
**SPOT-DISORDER (Hanson et al., 2017)**	1-2, 1159-1170, 1172-1173, 1611-1625, 1627-1628
**INTERPRO (Finn et al., 2017)**	Htt family domain: 1283-1999
**PONDR VLXT (Romero et al., 2001)**	204-219, 634-651, 1155-1178, 1318-1333, 1487-1502, 1815-1833, 1989-2002

Table S21. C-Score of the models of the ordered domains of an Htt-like protein identified in *C.elegans*

Ordered domains of an Htt-like protein	C-score
**Domain 1**	-1.26
**Domain 2**	0.04

Table S22. Structural homologs of domain 1 of the Htt-like protein in *C. elegans*, identified by I-TASSER

Rank	PDB hit	TM-score	RMSD (Å)	Identity (%)	Coverage
**1**	6EZ8	0.845	1.88	0.145	0.862
**2**	1Z3H	0.504	6.30	0.082	0.633
**3**	3W3T	0.497	6.86	0.075	0.637
**4**	3ND2	0.496	6.79	0.071	0.644
**5**	1QGK	0.484	6.71	0.054	0.626
**6**	6EMK	0.481	6.91	0.063	0.631
**7**	5YZ0	0.463	6.61	0.048	0.595
**8**	4XRI	0.459	7.32	0.068	0.621
**9**	4C0O	0.451	6.85	0.079	0.589
**10**	5VCH	0.438	7.17	0.070	0.582

Table S23. Structural homologs of domain 2 of the Htt-like protein in *C. elegans*, identified by I-TASSER

Rank	PDB hit	TM-score	RMSD (Å)	Identity (%)	Coverage
**1**	6EZ8	0.874	2.09	0.174	0.903
**2**	4KF7	0.576	6.32	0.061	0.767
**3**	5IJO	0.550	6.34	0.076	0.735
**4**	5HB4	0.543	6.27	0.070	0.725
**5**	5DLQ	0.534	7.40	0.061	0.772
**6**	3A6P	0.532	6.91	0.065	0.737
**7**	3GJX	0.530	6.76	0.065	0.733
**8**	5YZ0	0.523	6.12	0.051	0.692
**9**	3M1I	0.519	6.80	0.067	0.719
**10**	4KNH	0.503	6.86	0.062	0.711
